# Kidney and cystic volume imaging for disease presentation and progression in the cat autosomal dominant polycystic kidney disease large animal model

**DOI:** 10.1186/s12882-019-1448-1

**Published:** 2019-07-12

**Authors:** Yoshihiko Yu, Kate L. Shumway, Jodi S. Matheson, Marie E. Edwards, Timothy L. Kline, Leslie A. Lyons

**Affiliations:** 1 Department of Veterinary Medicine and Surgery, College of Veterinary Medicine, University of Missouri, Columbia, MO USA; 2 Laboratory of Veterinary Radiology, Nippon Veterinary and Life Science University, Tokyo, Japan; 3 Division of Nephrology and Hypertension, Mayo Clinic, Rochester, MN USA; 4 Department of Radiology, Mayo Clinic, Rochester, MN USA

**Keywords:** ADPKD, ESRD, Feline, *PKD1*

## Abstract

**Background:**

Approximately 30% of Persian cats have a c.10063C > A variant in *polycystin 1* (*PKD1*) homolog causing autosomal dominant polycystic kidney disease (ADPKD). The variant is lethal in utero when in the homozygous state and is the only ADPKD variant known in cats. Affected cats have a wide range of progression and disease severity. However, cats are an overlooked biomedical model and have not been used to test therapeutics and diets that may support human clinical trials. To reinvigorate the cat as a large animal model for ADPKD, the efficacy of imaging modalities was evaluated and estimates of kidney and fractional cystic volumes (FCV) determined.

**Methods:**

Three imaging modalities, ultrasonography, computed tomography (CT), and magnetic resonance imaging examined variation in disease presentation and disease progression in 11 felines with ADPKD. Imaging data was compared to well-known biomarkers for chronic kidney disease and glomerular filtration rate. Total kidney volume, total cystic volume, and FCV were determined for the first time in ADPKD cats. Two cats had follow-up examinations to evaluate progression.

**Results:**

FCV measurements were feasible in cats. CT was a rapid and an efficient modality for evaluating therapeutic effects that cause alterations in kidney volume and/or FCV. Biomarkers, including glomerular filtration rate and creatinine, were not predictive for disease progression in feline ADPKD. The wide variation in cystic presentation suggested genetic modifiers likely influence disease progression in cats. All imaging modalities had comparable resolutions to those acquired for humans, and software used for kidney and cystic volume estimates in humans proved useful for cats.

**Conclusions:**

Routine imaging protocols used in veterinary medicine are as robust and efficient for evaluating ADPKD in cats as those used in human medicine. Cats can be identified as fast and slow progressors, thus, could assist with genetic modifier discovery. Software to measure kidney and cystic volume in human ADPKD kidney studies is applicable and efficient in cats. The longer life and larger kidney size span than rodents, similar genetics, disease presentation and progression as humans suggest cats are an efficient biomedical model for evaluation of ADPKD therapeutics.

**Electronic supplementary material:**

The online version of this article (10.1186/s12882-019-1448-1) contains supplementary material, which is available to authorized users.

## Background

Autosomal dominant polycystic kidney disease (ADPKD) is one of the most commonly inherited disorders in humans, with an estimated prevalence of 1:400 to 1:1,000 [1]. ADPKD is the fourth most common cause of renal replacement therapy (i.e., dialysis or transplant) [2–4] and is generally diagnosed by imaging of the kidney using ultrasonography (US), computed tomography (CT), or magnetic resonance imaging (MRI). For ADPKD, approximately 75–85% of cases are caused by variants in *PKD1*, which encodes the protein known as polycystin-1 [5–7]. Most human families with ADPKD have novel variants and over 1,273 causal variants for *PKD1* are catalogued in the Autosomal Dominant Polycystic Kidney Disease Mutation Database: PKDB [8]. Because of this genetic heterogeneity, cohorts with the same *PKD1* variant or cohorts with the same genetic backgrounds are limited in humans, which inhibits the power of studies focused on genetic modifiers that would influence interfamilial, intrafamilial and sex differences in disease progression and responses to therapeutics. Genetic screening is also complicated in humans since *PKD1* includes 46 exons, has a large, ~ 14 kb mRNA [9] spanning a 47.2 kb genomic region, and six pseudogenes are present in the human genome [10, 11].

ADPKD is widely recognized as the most commonly inherited renal disease in the domestic cat, specifically cats of the Persian breed [12–16]. The feline *PKD1* variant (c.10063C > A) causes a stop codon at position 3284 in exon 29 (C3284X) [17] and is the only variant causing ADPKD in cats known to date. This variant is found in Persian-related breeds as well [17–19]. Hepatic and pancreatic cysts are present in some cats with ADPKD [14, 16, 20], however, hypertension is noted to be minor [21], and other vascular or systemic complications are not documented. Although many ADPKD cats remain subclinical through-out their lives, some show rapid disease progression, developing chronic kidney disease (CKD) secondary to ADPKD, and succumb to disease within 7 years of life or earlier, which is only mid-life for a cat [16, 22]. These younger cats that succumb to disease have consistent disease progressing to humans who have truncating *PKD1* variants [5]. Cats should be instrumental for identifying genetic modifiers of cystic progression and for deciphering variation in therapeutic responses since the solitary causal variant and control of the genetic background via colony of ADPKD cats will reduce variables in the analyses.

US, CT, and MRI are commonly used imaging modalities to evaluate disease progression and the therapeutic efficacy in humans with ADPKD [23], however, detailed and comparative imaging in feline ADPKD using different modalities is limited. US is routinely used to diagnose feline ADPKD, however, a study assessing the progression of the disease over time (approximately 1 year) showed an apparent improvement in a small number of cats [24], thus, the accuracy of US is questionable. In humans, US is used to screen ADPKD-suspected patients to follow changes over long periods of time, while CT and MRI are used to quantify changes in kidney parenchyma over shorter intervals [23]. In rodents, imaging accurately reflects kidney volumes, however, due to the small sizes of rodent kidneys, accurate evaluations of fractional cyst volume (FCV) are difficult and limited without the use of ultra-high field MRI (7 T and above) [25]. CT and MRI are the routine modalities used to evaluate the interventional efficacy of newly developed drugs in humans. MRI has not been used to evaluate ADPKD in cats, thus, baseline imaging studies could support the cats’ role for evaluating therapeutics.

Three imaging modalities, US, CT, and MRI, are used to examine variation in disease presentation and disease progression in feline ADPKD. Imaging was compared to well-known biomarkers for CKD, such as urine specific gravity (USG), blood urea nitrogen (BUN), serum creatinine (sCr), glomerular filtration rate (GFR) and symmetric dimethylarginine (SDMA), which is a novel biomarker for feline CKD at the earlier stage [26]. Total kidney volume (TKV), total cystic volume (TCV) and FCV were determined for the first time in ADPKD cats, demonstrating the wide variation in disease presentation, the potential identification of rapid and slow progression individual cats and the potential to evaluate therapeutic interventions.

## Methods

### Subjects

The cats represented an ADPKD cat colony housed at the University of Missouri (MU), which has been maintained for over 20 years. Five Persian cats with ADPKD were originally donated by private owners to establish the colony. All animal procedures were conducted in accordance with the National Research Council Guide for the Care and Use of Laboratory Animals and were approved by the MU Animal Care and Use Committee (protocol #8787). Cat housing and husbandry was overseen by MU Office of Animal Research. No cats were euthanized as part of this study. However, cats were euthanized by barbiturate overdose after sedation as dictated by poor health (renal failure). All cats were genotyped for the *PKD1* c.10063C > A that causes feline ADPKD (data not shown) [17, 27]. Diagnostic imaging studies were performed at MU during normal clinic hours (8:00 am – 6:00 pm) from November 2016 to May 2017, for the initial imaging, and June 2018 for the follow-up CT and MRI imaging of two cats. Cats were fasted for at least 12 h prior to diagnostic imaging. At the time of sedation for imaging, blood was collected by jugular venipuncture for a complete blood count, serum chemistry including BUN, sCr, SDMA. Urine was collected for urinalysis via cystocentesis using ultrasound guidance.

### Ultrasonography

US examinations were performed using an 8 MHz micro-convex transducer on a dedicated ultrasound unit (Logiq 9, GE Healthcare, Wauwatosa, WI, USA). Each kidney was scanned in sagittal and transverse planes by either a first-year veterinary radiology resident (K.L.S.) or a board-certified veterinary radiologist (J.S.M.). The length was measured, using internal digital calipers, as the longest point between the cranial and caudal poles in the sagittal plane. Width and height were measured in the transverse plane. TKV was calculated using the prolate ellipsoid formula [28].

### Glomerular filtration rate

GFRs were determined on the same day as the US imaging. Approximately 3 mCi of ^99^mTc-diethylenetriaminepentaacetic acid was administered intravenously. Images were obtained using a gamma camera (Equistand II, Diagnostic Services, Middlesex, NJ.) with a low energy all-purpose collimator and using a 256 × 256 matrix. The GFR was calculated for each kidney by a single observer (K.L.S.), using Mirage software and a previously described method for scintigraphic uptake [29].

### Magnetic resonance imaging

MRI was performed using a 3 T unit (Vantage Titan 3 T, Canon Medical Systems, Tustin, CA, USA) and a transmit/receive coil. Scans obtained included dorsal T2 (TR 2469–5675, TE 120) weighted imaging (T2WI) with 2 mm slices, a 0.2 mm interspace gap, matrix = 320 × 320–352, and number of acquisitions = 1–3. The scans were performed using respiratory gating to eliminate respiratory motion.

### Computed tomography

CT scans were performed using a third generation 64 slice instrument (Aquillion 64, Canon Medical Systems, Tustin, CA, USA) under the same anesthetic event as the MRI scan. Follow-up imaging was performed for two cats at 12 months and 15 months, which were thought to have fast and slow cyst progression (i.e., high and low FCV at the youngest age), respectively.

### Calculation of imaging parameters from CT and MRI data

TKVs were calculated from US as described above. TKVs as determined by CT and MRI was measured using a planimetry method with Fiji software common to veterinary practice [30]. To determine the area per slice, the outline of the kidney was traced by free-hand by a single investigator (K.L.S.) on each contiguous slice. Total volumes of the kidneys were obtained by summing the areas of the slices and multiplying by slice thickness. TKV and TCV were also calculated by a single analyst (M.E.E.) using the minimal interaction rapid organ segmentation (MIROS) method [31]. Cyst progression rate was predicted from the FCV per months (age) and from follow-up CT and MRI for two cats at 12 months and 15 months as described above.

### Statistical analysis

Means and standard deviations were calculated for 11 ADPKD cats for variables imaging indexes. All statistical analyses were performed using R software (version 3.3.3; R Foundation for Statistical Computing, Vienna, Austria). Comparisons of kidney volumes between male and female cats were analyzed using Mann–Whitney U test. The Spearman’s rank correlation test was used for the correlation analyses of imaging parameters and clinical data such as age, body weight and serum biomarkers. Kendall’s coefficient of concordance was calculated to evaluate the reliability between or among each modality. High agreement is indicated when Kendall’s coefficient of concordance (W) is higher than 0.75. *P* values < 0.05 were considered statistically significant. Data are expressed as mean ± standard deviation (SD).

## Results

### Signalment and renal biomarkers

Thirteen cats were examined, 11 cats were positive for the feline *PKD1* variant (c.10063C > A) (Table 1). The two normal cats were one male (17.88 months, 4.9 kg) and one female (15.12 months, 3.0 kg). All 13 cats had normal physical examinations at the time of imaging, including normal kidneys determined by palpation. A majority of cats were within normal limits for biomarkers, including USG, BUN, sCr, SDMA, and GFR (Table 1). Expected correlations were identified within the serum biomarkers including a positive correlation for SDMA and sCr (*r* = 0.88, *p* < 0.001), a positive correlation for sCr with BUN (*r* = 0.78, *p* = 0.004), and a positive correlation for SDMA with BUN (*r* = 0.61, *p* = 0.047).

**Table 1:** Signalment and renal biomarker data of ADPKD and control cats

Cat ID	Age^a^ (mo)	Weight (kg)	Sex	SDMA (0–14)	sCr (0.5–2.2)	BUN (19–35)	USG (1.035<)	GFR (2.5<)
Case 1	46.44	4.3	M	9	1.1	16	1.051	2.3
Case 2	97.08	3.9	F	15	1.6	24	1.028	2.78
Case 2^b^	~ 112	3.9	F	NA	2.4	36	1.015	NA
Case 2^b^	~ 116	2.7	F	NA	11.9	260	NA	NA
Case 3	19.44	3.6	F	12	1.2	24	1.055	3.95
Case 3^b^	~ 32	~ 3.6	F	14	1.3	24	1.046	NA
Case 4	33.72	3.4	F	9	1.2	22	1.06	2.6
Case 5	46.08	5.5	M	12	1.2	19	1.062	2.18
Case 6	47.04	4.2	M	9	0.9	13	1.06	3.12
Case 6^b^	~ 63	~ 4.2	M	8	0.7	18	1.062	
Case 7	33.36	5.2	F	9	1	18	1.064	2.15
Case 8	22.92	6.0	M	8	0.9	14	1.06	2.83
Case 9	58.68	4.7	M	4	0.9	16	1.066	3.3
Case 10	15.12	4.1	F	8	0.9	21	1.08	3.28
Case 11	16.44	4.8	M	8	0.9	13	1.056	2.64
mean ± SD	39.67 ± 23.89	4.52 ± 0.81	6F:7M	9.36 ± 2.84	1.07 ± 0.22	18.18 ± 4.14	1.058 ± 0.013	2.83 ± 0.55
Control 1	15.12	3	M	4	0.9	17	1.063	2.66
Control 2	17.88	4.86	F	8	0.9	24	1.066	2.97

^a^Age at time of imaging. *M* Male, *F* Female, *SDMA* Symmetric dimethylarginine (ug/dL), *sCr* serum creatinine (mg/dL), *BUN* Blood urea nitrogen (mg/dL), *USG* Urine specific gravity, *GFR* Glomerular filtration rate (ml/min/kg), *NA* Not available. ^b^Follow-up values were not used to calculate means ± SD

The oldest ADPKD cat (female; 8.1 years, Case 2) had an SDMA level of 15 μg/dL, sCr level of 1.6 mg/dL, and the lowest USG of 1.028. This cat was categorized as feline CKD Stage 2 based on the guidelines for feline CKD (International Renal interest Society; IRIS staging of CKD modified 2016; http://www.iris-kidney.com/guidelines/staging.html, accessed May 13, 2018). This cat was re-evaluated after 15 months and had mildly elevated levels of sCr (2.4 mg/dL) and BUN (36 mg/dL) and lower USG (1.015) indicating advancing renal failure. At 19 months after the initial imaging, this cat rapidly declined within 2 weeks, reducing from normal weight of 3.9 kg to 2.7 kg and was euthanized due to end stage renal disease. All other *APDKD* cats had cysts and were classified as feline CKD Stage 1.

### Glomerular flow rate

The two normal cats had GFRs of 2.66 and 2.97 ml/min/kg. The majority of ADPKD affected cats had normal average GFRs, ranging from 2.15–3.95 ml/min/kg. Three cats showed below normal GFRs of 2.30, 2.18 and 2.15 ml/min/kg (Table 1)**.** GFR had no suggested correlations with any biomarkers. GFR was not correlated with the TKV, regardless of modality used or the method of estimation of TKV (Additional file 1: Table S1). In addition, no correlation was identified between GFR and age of the cats (*r* = 0.1, *p* = 0.78) (Additional file 1: Figure S1).

### Imaging

**Fig. 1:**
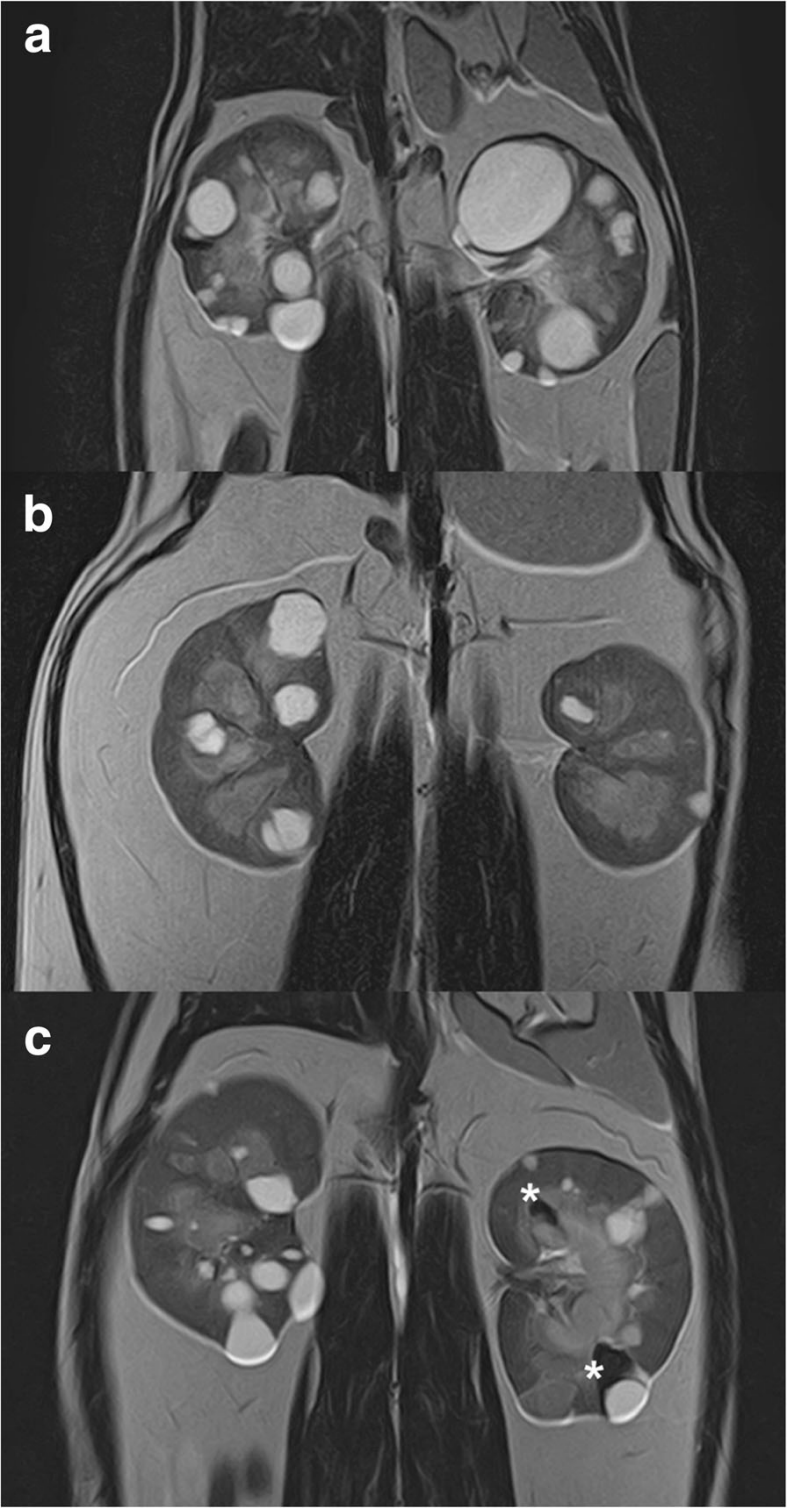
T2-weighted MR imaging of ADPKD cats. Cysts in ADPKD positive cats were T2-hyperintense. **a** T2-weighted imaging of 1.6 years old cat (Case 3) that shows high FCV. **b** T2WI of 1.9 years old cat (Case 8) that shows low FCV. **c** T2-weighted imaging of 4.9 years old cat (Case 9). T2-hypointense foci are seen, suggesting the presence of T2-hypointense material such as hemorrhage. This finding was observed in several cats

The normal cats did not have incidental cysts. A majority of cysts in the ADPKD cats were located in the cortex or at the corticomedullary junction, however, some cysts were located in the medulla, which is consistent with previous reports for feline ADPKD [32]. All ADPKD cats had bilateral, multiple cysts but with variation in number and size (Fig. 1). Simple cysts were homogeneously hyperintense on T2WI MRI, however, some cats showed some T2-hypointense foci (Fig. 1).

### Kidney volume estimates

Individual kidney volumes and TKVs were determined for each cat using three imaging modalities (Table 2**,** Additional file 1: Table S2). Males consistently had larger kidneys than females. The mean TKV ranged from 45.32 ± 13.50 ml for US to 69.39 ± 12.08 ml for planimetric MRI estimates. US-based volume estimates using the ellipsoid method were consistently the lowest estimated TKVs and MRI-based estimates were consistently the highest, especially the planimetric method for MRI. The TKV estimations for the two different methods were highly correlated within the CT and MRI modalities (*r* = 0.95–1, *p* < 0.01). US estimates were more strongly correlated with CT (MIROS) (*r* = 0.84, *p* < 0.01) than MRI (Additional file 1: Table S3). The ranks of the TKVs were consistent between planimetry and MIROS methods for MRI (W = 1, *p* = 0.042), and were fairly consistent between the planimetry and MIROS methods for CT (W = 0.92, *p* = 0.048) (Additional file 1: Table S4). The four cats with the lowest kidney volumes, including controls, were ranked more consistently across modalities than the cats with the larger kidney volumes. The cats with the three largest TKVs were ranked consistently between US and both MRI methods, although the US estimates were ~ 30% lower in volume. Approximately one-year post-imaging, TKVs for case 4 and case 5 were estimated by the water displacement method (data not shown) and were 26.5 and 51.2 ml, respectively (Additional file 1: Fig. S2).

**Table 2:** Summary of ADPKD cat individual and total kidney volumes

Volume	Modality (method)	All (*n* = 11)*		Male (*n* = 6)		Female (*n* = 5)*		*P*-value
		Mean	SD	Mean	SD	Mean	SD	
TKV	US (Ellipsoid)	45.32	13.50	51.49	6.87	37.92	16.46	0.2222
	CT (Planimetry)	56.52	17.79	66.63	9.72	44.38	18.33	0.0303
	CT (MIROS)	58.83	19.03	69.37	11.70	46.17	19.16	0.1255
	MRI (Planimetry)	69.37	11.30	71.06	12.99	66.00	7.96	NA
	MRI (MIROS)	62.91	10.36	64.71	11.41	59.32	8.63	NA
RKV	US (Ellipsoid)	21.91	6.93	25.72	4.64	17.33	6.72	0.08225
	CT (Planimetry)	27.54	9.87	34.07	6.06	19.71	7.55	0.008658
	CT (MIROS)	28.59	10.56	35.27	7.44	20.57	7.89	0.01732
	MRI (Planimetry)	34.67	7.81	36.90	7.65	30.21	7.28	NA
	MRI (MIROS)	30.89	7.08	33.35	6.71	25.96	5.79	NA
LKV	US (Ellipsoid)	23.41	7.15	25.77	2.95	20.59	9.93	0.9307
	CT (Planimetry)	28.97	8.82	32.56	4.52	24.67	11.25	0.329
	CT (MIROS)	30.24	9.40	34.10	5.15	25.61	11.77	0.1775
	MRI (Planimetry)	34.71	4.99	34.16	6.14	35.80	1.66	NA
	MRI (MIROS)	32.03	4.57	33.35	25.56	33.36	32.79	NA

*TKV* Total kidney volume, *RKV* Right kidney volume, *LKV* Left kidney volume, *NA* Not applicable. *all *n* = 9 for MRI, female *n* = 3 for MRI.

### Cystic index

TCVs were calculated using the MIROS method for both CT (n = 11) and MRI (*n* = 9) (Table 3, Additional file 1: Table S3). CT-based TCV ranged from 0.16 to 16.30 ml (mean = 5.79 ± 5.29 ml). MRI-based TCV ranged from 3.12 to 22.86 ml (mean = 9.83 ± 7.39 ml), ~ 70% larger than CT-based estimates. CT-based FCV ranged 0.63 to 28.22% (mean = 9.33 ± 8.37%). MRI-based FCV ranged from 6.18 to 44.64% (mean = 16.37 ± 14.06%), ~ 75% larger than the CT estimates. Differences between right and left kidney FCV ranged from 0.03 to 12.32% based on CT imaging and from 0.71 to 17.29% for MRI (Table 3). The average difference in FCV between left and right kidneys was 4.57% for CT-based estimates and 6.96% for MRI-based estimates. In comparisons to biomarkers, concentrations of sCr were mildly associated with FCVs (CT- or MRI-based) (*r* = 0.67, *p* = 0.02; *r* = 0.65, *p* = 0.06, respectively) and TCV (MRI-based) (*r* = 0.67, *p* = 0.05). Additionally, concentration of BUN was mildly associated with MRI-based TCV (*r* = 0.77, *p* = 0.02). GFR was not significantly correlated with TKV, TCV or FCV (Additional file 1: Table S1).

**Table 3:** TCV and FCV estimated from CT and MRI in ADPKD cats

		CT									MRI								
		TCV			FCV (%)			FCV/Mo (%)			TCV			FCV (%)			FCV/Mo (%)		
Cat ID	Age^a^ (mo)	Right	Left	Total	Right	Left	Total	Right	Left	Total	Right	Left	Total	Right	Left	Total	Right	Left	Total
Case 1	46.44	1.82	1.94	3.76	6.00	7.66	6.75	0.13	0.16	0.15	3.35	2.94	6.29	11.36	12.07	11.68	0.24	0.26	0.25
Case 2	97.08	7.55	8.75	16.30	33.65	24.76	28.22	0.35	0.26	0.29	10.04	12.82	22.86	49.26	41.58	44.64	0.51	0.43	0.46
Case 3	19.44	3.49	9.13	12.63	13.93	26.25	21.09	0.72	1.35	1.08	6.68	14.24	20.92	26.13	43.43	35.85	1.34	2.23	1.84
Case 4	33.72	0.91	0.90	1.81	7.09	7.18	7.13	0.21	0.21	0.21	NA								
Case 5	46.08	1.82	3.66	5.48	4.91	10.05	7.46	0.11	0.22	0.16	2.86	4.77	7.63	8.29	14.54	11.34	0.18	0.32	0.25
Case 6	47.04	0.29	2.52	2.82	1.18	7.74	4.91	0.03	0.16	0.10	0.72	3.99	4.71	2.47	11.82	7.49	0.05	0.25	0.16
Case 7	33.36	0.91	1.64	2.55	3.00	5.06	4.06	0.09	0.15	0.12	1.74	2.51	4.25	5.45	6.89	6.21	0.16	0.21	0.19
Case 8	22.92	2.87	0.37	3.24	7.53	1.09	4.50	0.33	0.05	0.20	5.23	0.69	5.92	13.09	2.05	8.05	0.57	0.09	0.35
Case 9	58.68	6.77	5.14	11.91	14.52	12.60	13.62	0.25	0.21	0.23	7.18	5.58	12.76	17.05	14.62	15.90	0.29	0.25	0.27
Case 10	15.12	0.07	0.08	0.16	0.61	0.64	0.63	0.04	0.04	0.04	NA								
Case 11	16.44	0.60	2.43	3.03	1.71	6.82	4.30	0.10	0.41	0.26	0.73	2.39	3.12	2.92	9.38	6.18	0.18	0.57	0.38
Average	39.67	2.46	3.32	5.79	8.56	9.99	9.33	0.21	0.29	0.26	4.28	5.55	9.83	15.11	17.28	16.37	0.39	0.51	0.46
SD	23.89	2.55	3.13	5.29	9.55	8.42	8.37	0.20	0.36	0.28	3.22	4.76	7.39	14.82	14.78	14.06	0.39	0.66	0.53

^a^Age in months at the initial scan. NA: Not available

### TKV, TCV and FCV progression

**Fig. 2:**
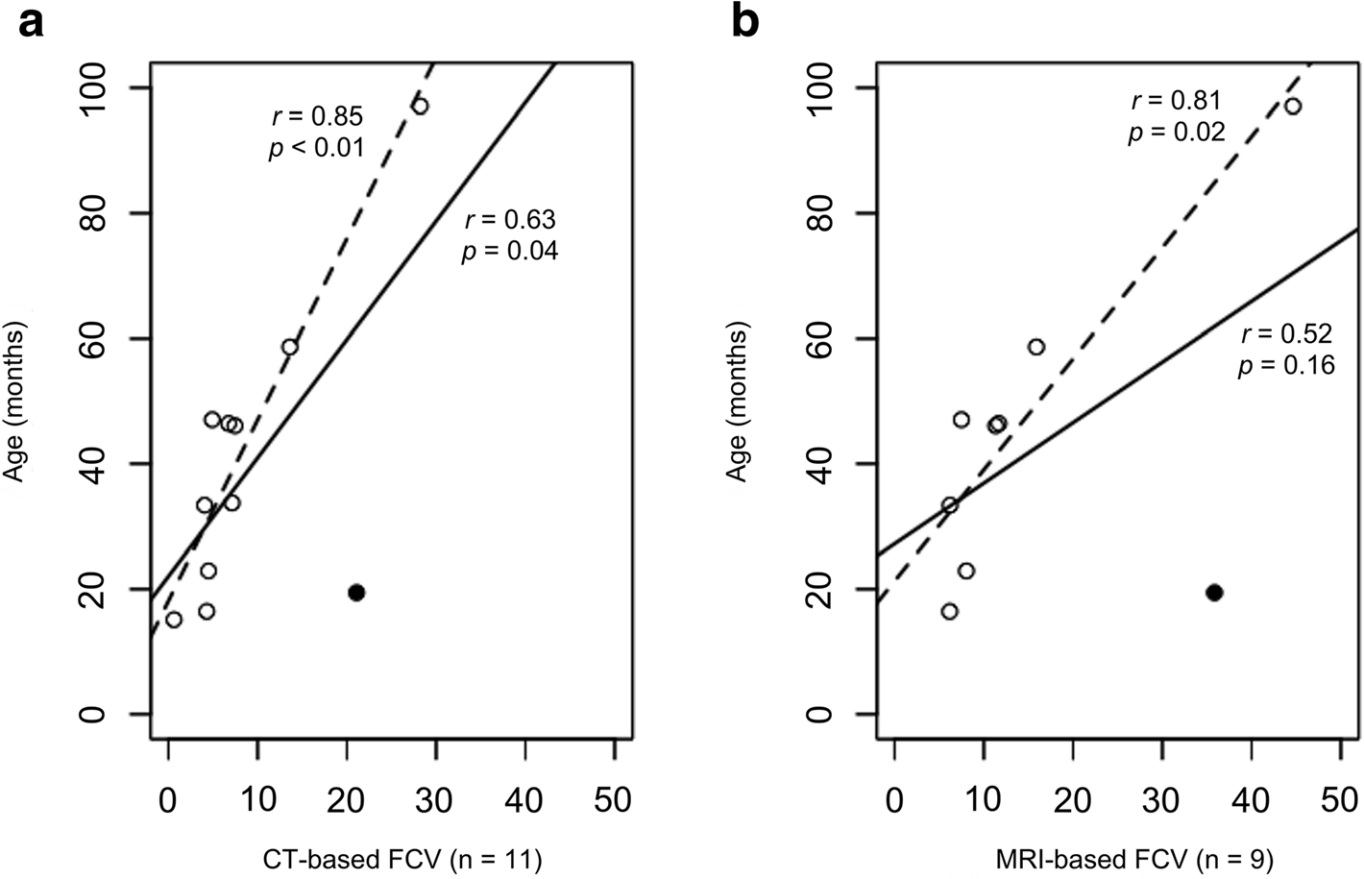
FCV correlation with age in feline ADPKD. **a** The graph illustrates significant positive correlation between CT-based FCV and age amongst 11 ADPKD cats (solid line). One cat is a suggested outlier (bold dots). When excluding this cat, the correlation increases (dashed line). **b** The graph demonstrates strong positive correlation between MRI-based FCV and age among eight ADPKD cats when the one outlier was excluded

FCV increased approximately 0.04 to 1.08% per month based on CT imaging and 0.16 to 1.84% per month based on MRI. The scatter plots for cat age versus FCV suggests cystic growth rate is linear during CKD Stage 1 of disease in cats (Fig. 2). However, one cat was a suggested “outlier” for cystic volume as this young female cat (Case 3, 19.45 months) had the second largest TCV and FCV (MRI and CT), or also, could also be considered as the high end of a range in disease progression. Removal of this “outlier” cat improved correlations (*r* > 0.8, *p* < 0.05) for both MRI-based and CT-based TCV and FCV estimates (Fig. 2).

**Fig. 3:**
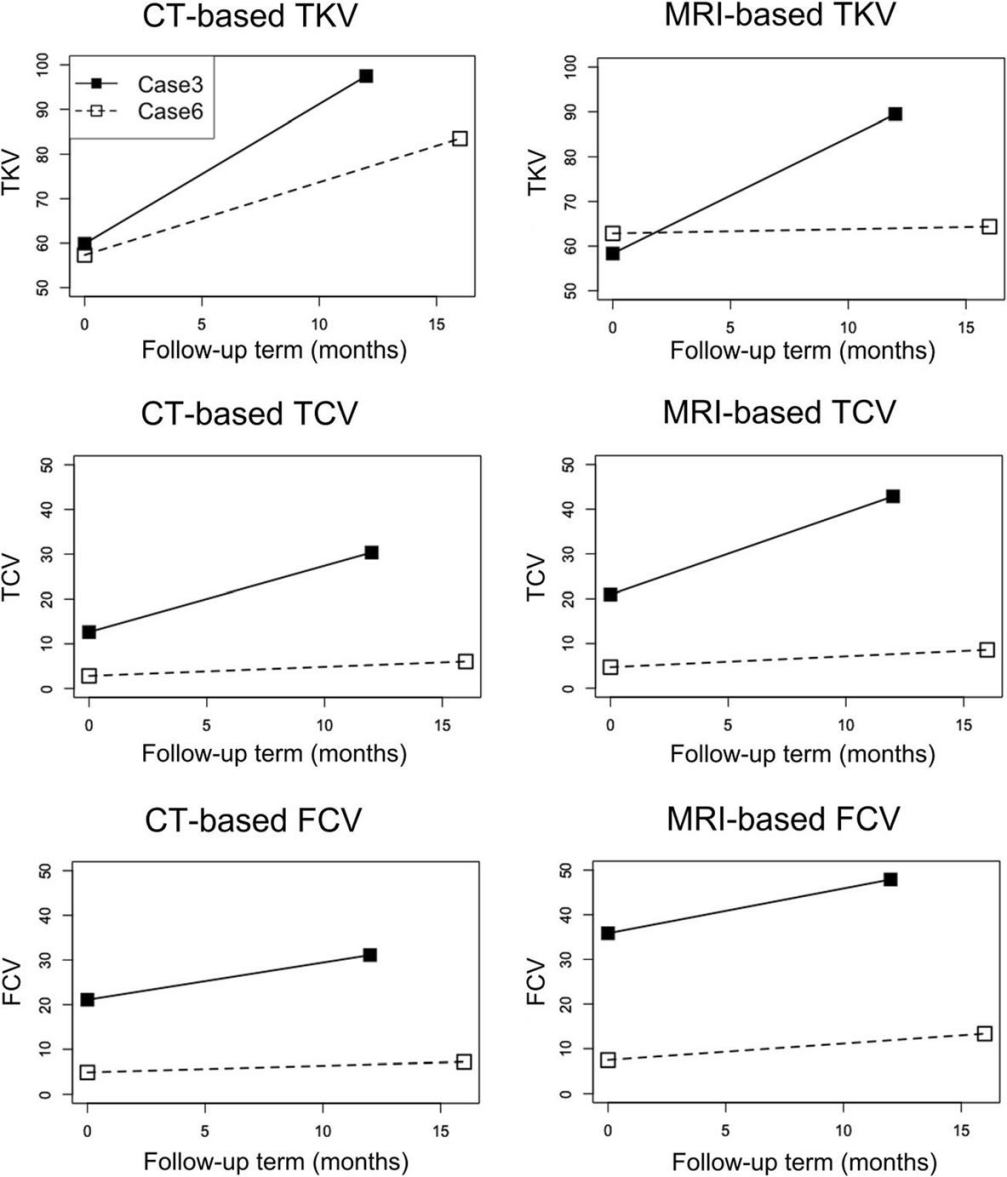
Longitudinal changes of imaging parameter using CT and MRI. Longitudinal changes were represented at the time of initial scan and follow-up scan using CT (left figures) and MRI (right figures). Data of the fast progression cat were demonstrated with solid lines and data of slow progression cat were demonstrated with broken lines. Note that increasing rate (slope) of FCV of two cats were similar in both CT and MRI

The general cyst progression rate (FCV per month) was an average of 0.19% per month (CT-based), if progression is considered linear, and 0.29% per month (MRI-based), when excluding the fastest outlier. The fast progression “outlier” cat (Case 3) had an expected FCV per month of 1.08% by CT and 1.84% by MRI. Case 6 was a slowly progressing cat with an expected FCV per month of 0.10% by CT and 0.16% by MRI. These two cats were re-evaluated after 12.6 and 15.0 months, respectively (Fig. 3, Table 4). TKV and TCV increased more drastically in the fast progression cat versus the slow progression cat and FCV increases were within the predicted estimates. However, the FCV increase based on MRI was counter intuitive as the estimate was 78.1% in the slow progression cat and only 33.62% for the fast progression cat. Also, the percent increase of FCV was similar between the two cats, 47.69 and 47.19% as estimated by CT. Based on the follow-up MRI (MIROS method), case 6 with the lowest FCV did not have a drastic increase in TKV (from 62.85 to 64.36 ml), however, had an 82.4% increase in TCV (4.71–8.59 ml) and a 78.1% increase of FCV (7.49–13.35%). Interestingly, percentage of monthly increase during the follow-up interval are within 9.8–11.9% in the both of cats, based on the both CT and MRI-based measurements.

**Table 4:** Renal volumetric changes in cats with ADPKD

Volume	Case	Modality	Initial	Follow up	Increase (ml)	Increase/mo. (ml)	Increase (%)	Monthly increase (%)	Initial FCV/mo	Expected increase
TKV	Fast 3	CT	59.88	97.48	37.60	2.98	62.79	12.92		
		MRI	58.35	89.49	31.14	2.47	53.37	12.78		
	Slow 6	CT	57.33	83.52	26.19	1.75	45.68	9.71		
		MRI	62.85	64.36	1.51	0.10	2.40	6.83		
TCV	Fast 3	CT	12.63	30.36	17.73	1.41	140.43	19.08		
		MRI	20.92	42.87	21.95	1.74	104.92	17.08		
	Slow 6	CT	2.82	6.04	3.22	0.21	114.43	14.30		
		MRI	4.71	8.59	3.88	0.26	82.38	12.16		
FCV	Fast 3	CT	21.09	31.14	10.06	0.80	47.69	11.72	1.08	13.61
		MRI	35.85	47.90	12.05	0.96	33.62	11.13	1.84	23.18
	Slow 6	CT	4.91	7.23	2.32	0.15	47.19	9.81	0.10	1.5
		MRI	7.49	13.35	5.85	0.39	78.10	11.87	0.16	2.4

Each estimate of TCV, FCV, and FCV per month were significantly correlated between modalities (i.e., CT-based and MRI-based) (*r* = 0.95–0.98, *p* < 0.01) (Additional file 1: Table S3). The rank of TCV was the same between the modalities except for one cat. Kendall’s coefficient of concordance showed W = 0.89 (*p* = 0.075). The rank of FCV showed W = 0.82 (*p* = 0.11) with Kendall’s coefficient of concordance (Additional file 1: Table S4).

## Discussion

ADPKD is caused by *PKD1* variants and is a common genetic and life-threatening disease for both humans and domestic cats. Although a variety of rodent models support PKD research, none support long-term trials of therapeutics. ADPKD domestic cats have a stop codon in exon 46, disrupting ~ 30% of polycystin-1 [17]. The disease is autosomal dominant, the homozygous state is lethal in utero, and cysts develop prior 8 months of age. Thus, this cat models mimics the human condition genetically. However, little is known about the changes in kidney imaging parameters in ADPKD cats, especially TKV, TCV, and FCV.

The rate and extent of cystic progression has not been examined in cats, although generally, cysts worsen as the cat ages. The average life span of cats is ~ 13–17 years of age and many die of CKD [33, 34]. One of the factors leading to the discovery of ADPKD was early CKD as cats were dying at 3–4 years old. However, many cats with ADPKD live a normal life span. Thus, the variation in disease severity and rate of progression is recognized but undocumented. Imaging is required to determine disease severity and to monitor progression even though a genetic test exists for feline ADPKD [17, 27]. The range in severity suggests additional genetic and non-genetic factors influence disease progression.

Eleven ADPKD cats were examined by different modalities to quantify the variation in the cats and to compare imaging modalities. Ten of eleven cats had no suggestion of renal compromise other than the presence of cysts, thus renal biomarkers, including SDMA, sCr and BUN, are not predictive risk indicators of feline ADPKD and disease severity. GFR was also not indicative of kidney disease in the ADPKD cats. Significant parenchymal loss is likely to be required before abnormal GFRs are observed in APDKD cats [32]. The GFRs were within normal limits or low for the ADPKD cats, including the oldest cat and five ADPKD cats with ~ 15% FCV. Three cats with lower FCV had mildly lowered GFRs, which may be partially due to reduced renal artery pressure secondary to the anesthesia. The age of the cat cohort is relatively young (mean: 39.7 months [range: 16.4–97.1 months]. Many human ADPKD patients have no obvious clinical symptoms until the third or fourth decade of life [35] and renal function usually remains normal until the fourth to sixth decade of life [36]. A recent study of 377 cats from Japan indicated of cats with the cat *PKD1* mutation, the incidence of a high concentration of plasma Cre (>1.6 mg/dl: ≥IRIS-CKD stage 2) was greater in cats older than 3 years old, and especially in those older than 7 years. In contrast, a few cats aged ≥9 years had low plasma Cre concentrations (≤1.6 mg/dl) [37]. Therefore, the correlation between TKV and GFR may improve in older ADPKD cats.

TKV, rather than renal function, is suggested as the more appropriate biomarker for monitoring and predicting disease progression in human medicine [38]. In humans, TKV measurements are obtained by MRI or CT to assess the efficacy of therapeutic interventions [23, 39, 40]. US, CT and MRI are all minimally invasive and highly diagnostic for ADPKD in cats, with US being the most rapid, least expensive, and most accessible. MRI T2WI is sensitive and sufficient for volume measurement and CT is associated with radiation exposure, hence CT is less favored in human medicine [2, 40]. However, CT is favored in cats due to decreased anesthesia requirements and the minimal concern of long-term consequences of radiation exposure.

US, CT and MRI were conducted in ADPKD cats to estimate TKV. Normal cat kidney volumes have been estimated using water displacement at 18.99 ± 7.68 cm^3^, and US and CT imaging from 14.8 ± 2.9 ml to 19.01 ± 7.55 ml, respectively [32, 41]. For ADPKD cats, total kidney volumes have been estimated as 27.4 ± 10.3 ml using US and 29.3 ± 13.4 ml by CT [32], ~ 30% larger than normal cat kidneys. In this study, the 11 ADPKD cats had kidney volume estimates from 23.0 ± 6.91 ml using US to as high as 34.69 ± 6.36 ml using the planimetric method for MRI thereby overlapping within normal limits but also have significantly larger TKVs. Overall, US and planimetry CT TKV estimates were slightly smaller, however, within the ranges of previous study. The cats in the previous study (mean age: 59 ± 10 months) [32] were an average of 20 months older than this study (mean age: 39.67 ± 23.89 months), thus would be expected to have larger TKVs. The TKV estimates for a given cat were increasing larger over US-based TKV estimates by 20, 22, 27, 34%, estimating by planimetry CT, MIROS CT, MIROS MRI, and planimetry MRI, respectively. The TKV for case 4 that was estimated by water displacement was consistent with CT estimates, but case 5 estimates were over-estimated by CT and MRI, potentially due to the adipose tissue in this overweight cat.

In one study, cyst growth in humans is reported as relatively symmetrically and at a steady rate [42]. However, other research has indicated variable expression of cyst progression, even in the same family [43, 44]. In the cats, FCVs were highly correlated with age, supporting cyst size increasing with age and a steady rate. However, Case 3 showed 1.08 and 1.84% increase in FCV per month, by CT and MRI, respectively, which is approximately six times the average increase than the other ADPKD cats. Once this “outlier” was removed from the correlation analysis, FCV per month showed an average of 0.19 and 0.29% by CT and MRI, respectively, and was more highly correlated with age. Thus, for cats, most cats have a consistent rate of disease progression, however, some cats are highly variability. Furthermore, the value on TCV and FCV showed wide ranges as shown in Table 3. Although ages were not uniform, these findings also indicated that variability of cyst progression speed among feline ADPKD cats, regardless of genetic homogeneity for the mutation. Feline ADPKD is individually variable between kidneys of a given cat, even though all cats had the same germline mutation (*PKD1* c.10063C > A), suggesting other factors modify disease expression and progression.

Cats as a large animal model are clearly an asset to evaluate the efficacy and safety of the development of drugs and gene therapies [45, 46]. Although the exact same mutation is not found in cats and humans, cats and humans have similarly disruptive mutations that truncate approximately 30% of polycystin-1. Feline ADPKD may fill a void of translational research between rodent and human ADPKD. The rodent models do not perfectly recapitulate human ADPKD in terms of differences in lifespan, metabolism, and renal anatomy [47]. Feline ADPKD has the potential to overcome these differences. Although feline ADPKD is caused by a single *PKD1* c.10063C > A, considering progression variability, disease progression of feline ADPKD is likely influenced by other genetic and/or environmental factors, such as those identified in humans [48–51]. In addition, cats could support studies focusing on pleiotrophic effects of ADPKD. A recent study showed co-occurrence of hepatic and renal cysts was found in 20 (12.6%) out of 159 cat cases with renal cyst(s), and all cases were positive for the *PKD1* mutation [37]. Identification of genetic modifiers could lead to selection of appropriate cats with ADPKD for therapeutic trials and reducing animal use and improving study design.

## Conclusions

In conclusion, TKV, TCV, and FCV estimations are valuable for evaluating disease status of feline ADPKD and could lead to renal failure risk classifications analogous to humans [52, 53 ]. The current study shows imaging-based estimations for TKV, TCV, FCV, and FCV per month, can be easily determined for cats with ADPKD using CT and MRI. While MRI is preferred to CT in human medicine due to radiation exposure, CT is more practical to evaluate disease progression for feline ADPKD because of rapid image acquisition that only requires sedation or light anesthesia, lower cost, and increasing availability. This study also demonstrates that cystic progression is individual and variable in cats and since cats have only on ADPKD variant and the genetic model is highly similar to humans, cats should be useful for studies focused on genetic modifiers and efficacy of therapeutics.

## Additional file

Additional file 1: Supplementary Methods. **Table S1.** Correlation of CKD biomarkers and GFRs with total kidney volume. **Table S2.** Estimations of individual kidney volumes and TKVs for each ADPKD and control cat. **Table S3.** Correlation of kidney imaging parameters by imaging modalities. **Table S4.** Rank of kidney volumes estimated by different modalities. **Figure S1**.GFRs at different ages in 11 cats with ADPKD. **Figure S2.** Post-mortem kidneys of cats with ADPKD. (DOCX 1170 kb)

## Data Availability

All data generated or analyzed during this study are included in this published article [and its supplementary information files]. Raw data files of imaging are available from the corresponding author on reasonable request.

## Abbreviations

ADPKD: Autosomal dominant polycystic kidney disease
BUN: Blood urea nitrogen
CKD: Chronic kidney disease
CT: Computed tomography
FCV: Fractional cystic volume
GFR: Glomerular filtration rate
IRIS: International Renal Interest Society
LKV: Left kidney volume
MIROS: Minimal interaction rapid organ segmentation
MRI: Magnetic resonance imaging
mRNA: Messenger RNA
MU: University of Missouri
PKD: Polycystic kidney disease
*PKD1*: *Polycystin 1*
PKDB: Polycystic Kidney Disease Mutation Database
RKV: Right kidney volume
sCr: Serum creatinine
SD: Standard deviation
SDMA: Symmetric dimethylarginine
TCV: Total cystic volume
TKV: Total kidney volume
US: Ultrasonography
USG: Urine specific gravity
