# Supplementary material for: Kidney and cystic volume imaging for disease presentation and progression in the cat autosomal dominant polycystic kidney disease large animal model
Source: BMC Nephrol. 2019 Jul 12;20:259. doi: 10.1186/s12882-019-1448-1 (PMC6625046; doi:10.1186/s12882-019-1448-1)
Supplement: Supplementary file 1 — Supplementary Methods. Table S1. Correlation of CKD biomarkers and GFRs with total kidney volume. Table S2. Estimations of individual kidney volumes and TKVs for each ADPKD and control cat. Table S3. Correlation of kidney imaging parameters by imaging modalities. Table S4. Rank of kidney volumes estimated by different modalities. Figure S1.GFRs at different ages in 11 cats with ADPKD. Figure S2. Post-mortem kidneys of cats with ADPKD. (DOCX 1170 kb) [file 12882_2019_1448_MOESM1_ESM.docx]

**Additional file 1**

**Supplementary Methods**

**Table S1. Correlation of CKD biomarkers and GFRs with total kidney volume.**

**Table S2. Estimations of individual kidney volumes and TKVs for each ADPKD and control cat.**

**Table S3. Correlation of kidney imaging parameters by imaging modalities.**

**Table S4. Rank of kidney volumes estimated by different modalities.**

**Figure S1. GFRs at different ages in 11 cats with ADPKD.**

**Figure S2**. **Post-mortem kidneys of cats with ADPKD.**

**Supplementary Methods**

**Ultrasonography**

The US examinations were performed using an 8 MHz micro-convex transducer on a dedicated ultrasound unit (Logiq 9, GE Healthcare, Wauwatosa, WI, USA). The cats were sedated with a combination of buprenorphine (0.01mg/kg) and alfaxalone (5mg/kg) administered intramuscularly. An intravenous catheter was placed and exams were performed in dorsal recumbency. Each kidney was scanned in sagittal and transverse planes by either (a first-year veterinary radiology resident (K.L.S.) or a board-certified veterinary radiologist (J.S.M.). Three measurements (length, width, and height) were obtained to calculate total kidney volume. The length was measured, using internal digital calipers, as the longest point between the cranial and caudal poles in the sagittal plane. Width and height were measured in the transverse plane. TKV was calculated using the prolate ellipsoid formula.^1^ Each kidney was measured three times and the average volume measurement was used for analysis.

**Glomerular Filtration Rate**

GFRs were determined on the same day as the US imaging. Approximately 3mCi of ^99^mTc-diethylenetriaminepentaacetic acid (DTPA) was administered intravenously. Images were obtained using a gamma camera (Equistand II, Diagnostic Services, Middlesex, NJ.) with a low energy all-purpose collimator and using a 256 x 256 matrix. The cats were placed in left lateral recumbency with the dorsum in contact with the gamma camera, which was positioned vertically. Images were captured every three seconds over a four minutes period and complied to form a single image ensuring that there was no movement by the cat outside of normal respiratory motion. Static pre- and post- syringe counts were obtained over a 60 second period and compiled to form a single image. The GFR was calculated for each kidney by a single observer (K.L.S.), Using Mirage software and a previously described method for scintigraphic uptake.^2^ The cats were then kept in radiation isolation for a minimum of 60 hrs prior to CT and MRI scans. GFRs > 2.5 ml/min/kg are considered normal in adult cats. ^2^

**Magnetic Resonance Imaging**

MRI was performed using a 3T unit (Vantage Titan 3T, Canon Medical Systems, Tustin, CA, USA) and a transmit/receive coil. Scans obtained included dorsal T2 (TR 2469-5675, TE 120) weighted imaging (T2WI) with 2 mm slices, a 0.2 mm interspace gap, matrix = 320×320–352, and number of acquisition = 1–3. The cats were placed under general anesthesia using anesthetic protocols deemed appropriate by the anesthesia service of the MU Veterinary Health Center. All cats were intubated and anesthesia was maintained using isoflurane gas. The cats were placed in dorsal recumbency and the scans were performed using respiratory gating to eliminate respiratory motion.

**Computed Tomography**

CT scans were performed using a third generation 64 slice instrument (Aquillion 64, Canon Medical Systems, Tustin, CA, USA) under the same anesthetic event as the MRI scan. The scan was performed using a third generation 16 slice instrument (Celesteion, Canon Medical systems, Tustin, CA, USA) for one cat because of the institutional issue. Each cat was placed in dorsal recumbency and pre- and post-contrast images were obtained from the diaphragm through the anus (slice thickness 1–2 mm, kvp 80–100, mAs 230–500). Intravenous non-ionic, iodinated contrast media (Omnipaque 350 Iohexol injection, GE Healthcare, Marlboro, MA, USA) was administered at a dose of 0.5 mg/kg and post-contrast images were obtained after a three-minute delay. Follow-up imaging was performed for two cats at 12 months and 15 months, which were thought to have fast and slow cyst progression (i.e., high and low FCV at the youngest age), respectively.

**References**

1. Nyland, TG, Fisher, PE, Gregory, CR, Wisner, ER: Ultrasonographic evaluation of renal size in dogs with acute allograft rejection. *Vet Radiol Ultrasound,* 38**:** 55-61, 1997.
2. Daniel, GB, Mitchell, SK, Mawby, D, Sackman, JE, Schmidt, D: Renal nuclear medicine: a review. *Vet Radiol Ultrasound,* 40**:** 572-587, 1999.

**Table S1. Correlation of CKD biomarkers and GFRs with total kidney volume.**

|  | SDMA | Creatinine | BUN | USG | GFR |
| --- | --- | --- | --- | --- | --- |
| US-based TKV | -0.23 (0.49) | -0.20 (0.55) | -0.22 (0.51) | 0.06 (0.87) | 0.18 (0.59) |
| CT-based TKV (Planimetry) | -0.26 (0.44) | -0.33 (0.33) | -0.52 (0.10) | 0.22 (0.52) | 0.04 (0.92) |
| CT-based TKV (MIROS) | -0.19 (0.58) | -0.16 (0.63) | -0.32 (0.34) | 0.17 (0.61) | -0.02 (0.96) |
| MRI-based TKV (Plainmetry) | -0.44 (0.24) | -0.38 (0.32) | -0.05 (0.90) | 0.83 (0.01) | 0.13 (0.73) |
| MRI-based TKV (MIROS) | -0.44 (0.24) | -0.38 (0.32) | -0.05 (0.90) | 0.83 (0.01) | 0.13 (0.73) |
| CT-based TCV (MIROS) | 0.42 (0.19) | 0.45 (0.16) | 0.26 (0.45) | -0.52 (0.10) | 0.24 (0.48) |
| CT-based FCV (MIROS) | 0.56 (0.08) | 0.67 (0.02) | 0.50 (0.11) | -0.48 (0.14) | 0.24 (0.48) |
| MRI-based TCV (MIROS) | 0.52 (0.15) | 0.67 (0.05) | 0.77 (0.02) | -0.31 (0.42) | 0.43 (0.24) |
| MRI-based FCV (MIROS) | 0.48 (0.19) | 0.65 (0.06) | 0.73 (0.03) | -0.39 (0.29) | 0.45 (0.22) |

Spearman's correlation coefficient r (p-value) is represented.

**Table S2. Estimations of individual kidney volumes and TKVs for each ADPKD and control cat.**

| **Cat ID** |  | **Sex** | **US (Ellipsoid formula)** | | | **CT (planimetry method)** | | | **CT (MIROS method)** | | | **MRI (planimetry method)** | | | **MRI (MIROS method)** | | |
| --- | --- | --- | --- | --- | --- | --- | --- | --- | --- | --- | --- | --- | --- | --- | --- | --- | --- |
|  | **Wt (Kg)** |  | **RKV** | **LKV** | **TKV** | **RKV** | **LKV** | **TKV** | **RKV** | **LKV** | **TKV** | **RKV** | **LKV** | **TKV** | **RKV** | **LKV** | **TKV** |
| **Case 1** | 4.3 | M | 24 | 20.85 | 44.85 | 29.89 | 25.12 | 55.01 | 30.36 | 25.27 | 55.63 | 31.95 | 25.69 | 57.64 | 29.50 | 24.36 | 53.86 |
| **Case 2** | 3.9 | F | 19.37 | 28.67 | 48.04 | 21.37 | 33.74 | 55.12 | 22.44 | 35.33 | 57.77 | 22.69 | 34.45 | 57.13 | 20.38 | 30.83 | 51.21 |
| **Case 3** | 3.6 | F | 23.28 | 26.28 | 49.56 | 24.25 | 33.75 | 58.00 | 25.08 | 34.80 | 59.88 | 30.71 | 37.65 | 68.36 | 25.56 | 32.79 | 58.35 |
| **Case 4** | 3.4 | F | 10.41 | 10.05 | 20.46 | 12.69 | 12.49 | 25.18 | 12.82 | 12.56 | 25.38 | NA | NA | NA | NA | NA | NA |
| **Case 5*** | 5.5* | M | 23.36 | 24.42 | 47.78 | 35.20 | 34.79 | 69.98 | 36.99 | 36.42 | 73.41 | 36.87 | 34.84 | 71.70 | 34.50 | 32.81 | 67.31 |
| **Case 6** | 4.2 | M | 21.22 | 26.14 | 47.36 | 27.49 | 32.93 | 60.41 | 24.72 | 32.61 | 57.33 | 32.12 | 37.12 | 69.25 | 29.1 | 33.75 | 62.85 |
| **Case 7** | 5.2 | F | 23.61 | 28.47 | 52.08 | 28.93 | 31.05 | 59.99 | 30.31 | 32.36 | 62.67 | 37.22 | 35.30 | 72.52 | 31.94 | 36.45 | 68.39 |
| **Case 8** | 6.0 | M | 32.13 | 27.98 | 60.11 | 36.27 | 31.10 | 67.37 | 38.11 | 33.87 | 71.98 | 45.92 | 37.16 | 83.08 | 39.96 | 33.59 | 73.55 |
| **Case 9** | 4.7 | M | 31.03 | 29.28 | 60.31 | 44.46 | 38.84 | 83.31 | 46.66 | 40.78 | 87.44 | 46.32 | 42.03 | 88.34 | 42.10 | 38.16 | 80.26 |
| **Case 10** | 4.1 | F | 10 | 9.46 | 19.46 | 11.31 | 12.32 | 23.63 | 12.19 | 12.98 | 25.17 | NA | NA | NA | NA | NA | NA |
| **Case 11** | 4.8 | M | 22.59 | 25.92 | 48.51 | 31.10 | 32.60 | 63.69 | 34.79 | 35.63 | 70.42 | 28.22 | 28.12 | 56.33 | 24.96 | 25.49 | 50.45 |
| **Control 1** | 3 | M | 16.38 | 14.93 | 31.31 | 21.429 | 20.749 | 42.178 | 22.83 | 22.35 | 45.18 | 23.535 | 21.622 | 45.157 | 22.83 | 22.35 | 45.18 |
| **Control 2** | 4.86 | F | 11.97 | 12.66 | 24.63 | 15.99 | 16.19 | 32.18 | 16.85 | 17.22 | 34.07 | NA | NA | NA | NA | NA | NA |

NA: Not available *Body condition score of 7 – 8, overweight.

**Table S3. Correlation of kidney imaging parameters by imaging modalities.**

|  | TKV |
| --- | --- |
| US – CT (planimetry) | 0.73 (<0.01) |
| US – CT (MIROS) | 0.84 (<0.01) |
| US – MRI (planimetry) | 0.62 (0.08) |
| US – MRI (MIROS) | 0.62 (0.08) |
| CT (planimetry) – CT (MIROS) | 0.95 (<0.01) |
| CT (planimetry) – MRI (planimetry) | 0.63(0.07) |
| CT (planimetry) – MRI (MIROS) | 0.63(0.07) |
| MRI (planimetry) – CT (MIROS) | 0.6 (0.09) |
| MRI (planimetry) – MRI (MIROS) | 1 (<0.01) |
| CT (MIROS method) – MRI (MIROS method) | 0.6 (0.09) |
|  | TCV |
| CT (MIROS method) – MRI (MIROS method) | 0.95 (<0.01) |
|  | FCV |
| CT (MIROS method) – MRI (MIROS method) | 0.95 (<0.01) |
|  | FCV/month |
| CT (MIROS method) – MRI (MIROS method) | 0.98 (<0.01) |

Values were represented as Spearman's rank correlation coefficient (*p*-value). Since the difference of sample number for US or CT (n = 11) and MRI (n = 9), comparisons between US or CT and MRI were performed with samples that underwent CT and MRI.

**Table S4. Rank of kidney volumes estimated by different modalities.**

| Cat ID | US (Ellipsoid formula) | | |  | CT (planimetry method) | | |  | CT (MIROS method) | | |  | MRI (planimetry method) | | |  | MRI (MIROS method) | | |  |
| --- | --- | --- | --- | --- | --- | --- | --- | --- | --- | --- | --- | --- | --- | --- | --- | --- | --- | --- | --- | --- |
|  | RKV | LKV | TKV | R | RKV | LKV | TKV | R | RKV | LKV | TKV | R | RKV | LKV | TKV | R | RKV | LKV | TKV | R |
| Case 10 | 10 | 9.46 | 19.46 | 1 | 11.31 | 12.32 | 23.63 | 1 | 12.19 | 12.98 | 25.17 | 1 | NA | | | | | | | |
| Case 4 | 10.41 | 10.05 | 20.46 | 2 | 12.69 | 12.49 | 25.18 | 2 | 12.82 | 12.56 | 25.38 | 2 | NA | | | | | | | |
| Control 2 | 11.97 | 12.66 | 24.63 | 3 | 15.99 | 16.19 | 32.18 | 3 | 16.85 | 17.22 | 34.07 | 3 | NA | | | | | | | |
| Control 1 | 16.38 | 14.93 | 31.31 | 4 | 21.429 | 20.749 | 42.18 | 4 | 22.83 | 22.35 | 45.18 | 4 | 23.535 | 21.622 | 45.16 | 4 | 22.83 | 22.35 | 45.18 | 4 |
| Case 1 | 24 | 20.85 | 44.85 | 5 | 29.89 | 25.12 | 55.01 | 5 | 30.36 | 25.27 | 55.63 | 5 | 31.95 | 25.69 | 57.64 | 7 | 29.50 | 24.36 | 53.86 | 7 |
| Case 6 | 21.22 | 26.14 | 47.36 | 6 | 27.49 | 32.93 | 60.41 | 9 | 24.72 | 32.61 | 57.33 | 6 | 32.12 | 37.12 | 69.25 | 9 | 29.1 | 33.75 | 62.85 | 9 |
| Case 5* | 23.36 | 24.42 | 47.78 | 7 | 35.20 | 34.79 | 69.98 | 12 | 36.99 | 36.42 | 73.41 | 12 | 36.87 | 34.84 | 71.70 | 10 | 34.50 | 32.81 | 67.31 | 10 |
| Case 2 | 19.37 | 28.67 | 48.04 | 8 | 21.37 | 33.74 | 55.12 | 6 | 22.44 | 35.33 | 57.77 | 7 | 22.69 | 34.45 | 57.13 | 6 | 20.38 | 30.83 | 51.21 | 6 |
| Case 11 | 22.59 | 25.92 | 48.51 | 9 | 31.10 | 32.60 | 63.69 | 10 | 34.79 | 35.63 | 70.42 | 10 | 28.22 | 28.12 | 56.33 | 5 | 24.96 | 25.49 | 50.45 | 5 |
| Case 3 | 23.28 | 26.28 | 49.56 | 10 | 24.25 | 33.75 | 58.00 | 7 | 25.08 | 34.80 | 59.88 | 8 | 30.71 | 37.65 | 68.36 | 8 | 25.56 | 32.79 | 58.35 | 8 |
| Case 7 | 23.61 | 28.47 | 52.08 | 11 | 28.93 | 31.05 | 59.99 | 8 | 30.31 | 32.36 | 62.67 | 9 | 37.22 | 35.30 | 72.52 | 11 | 31.94 | 36.45 | 68.39 | 11 |
| Case 8 | 32.13 | 27.98 | 60.11 | 12 | 36.27 | 31.10 | 67.37 | 11 | 38.11 | 33.87 | 71.98 | 11 | 45.92 | 37.16 | 83.08 | 12 | 39.96 | 33.59 | 73.55 | 12 |
| Case 9 | 31.03 | 29.28 | 60.31 | 13 | 44.46 | 38.84 | 83.31 | 13 | 46.66 | 40.78 | 87.44 | 13 | 46.32 | 42.03 | 88.34 | 13 | 42.10 | 38.16 | 80.26 | 13 |

R is the rank order of volume. NA: Not available *Body condition score of 7 – 8, overweight.

**
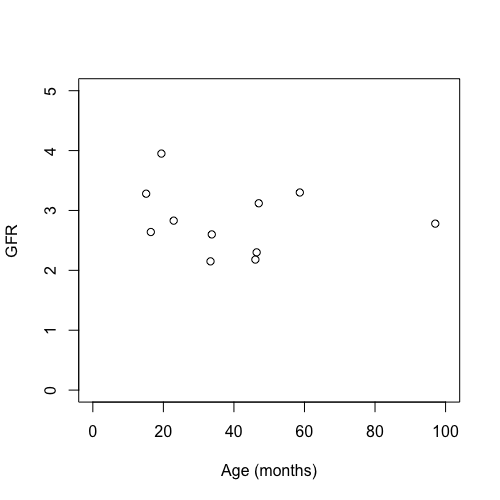
**

**Figure S1**. GFRs at different ages in 11 cats with ADPKD.

GFRs did not have a significant correlation with age of cat for this study.


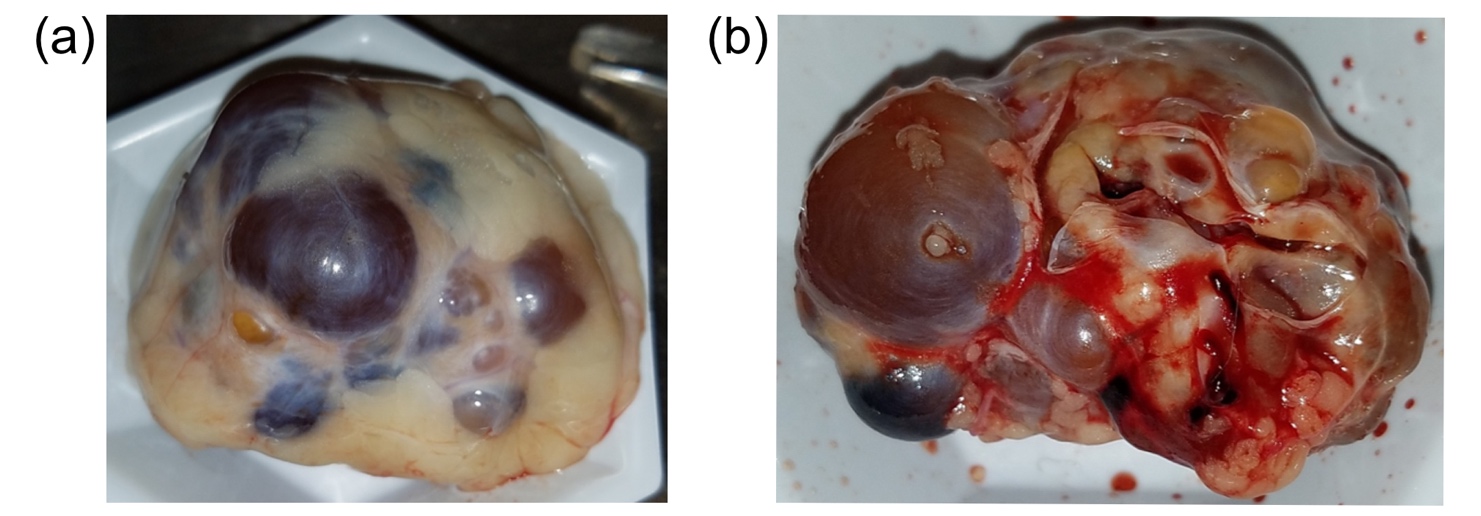


**Figure S2**. Post-mortem kidneys of cats with ADPKD. (a) Case 4’s kidney. Water displacement was used to evaluate kidney volumes for ADPKD cat kidneys post-mortem approximately one-year post imaging. a) Case 4 TKV was 26.5 ml with imaging estimates ranging from 20.46 – 25.38 ml. (b) Case 5’s kidney. TKV was 51.2 ml with imaging estimates ranging from 47.78 – 73.41 ml – cat was overweight with extensive adipose tissue surrounding the kidneys.
